# Three-dimensional evaluation of dentigerous cysts in the Turkish subpopulation

**DOI:** 10.1186/s12903-024-04448-7

**Published:** 2024-06-10

**Authors:** İlknur Eninanç, Esra Mavi

**Affiliations:** 1 Department of Oral and Maxillofacial Radiology, Faculty of Dentistry, Sivas Cumhuriyet University, Sivas, Turkey; 2 Department of Oral and Maxillofacial Surgery, Faculty of Dentistry, Sivas Cumhuriyet University, Sivas, Turkey

**Keywords:** Dentigerous cyst types, Multiple cysts, Marsupialization, Enucleation

## Abstract

**Background:**

To investigate the radiological and demographic features, types, distribution, and treatment methods of dentigerous cysts (DC).

**Methods:**

Panoramic radiographs and cone beam computed tomography (CBCT) images of patients diagnosed with DC based on biopsy results between January 2020 and December 2023 were examined. In patients from different age groups, the numbers, types and locations, and radiological features of DCs, associated changes in surrounding tissues, and treatment methods used were reviewed.

**Results:**

Among 95 patients with DC (66 males, 29 females), sex and age distributions were comparable between those with a single cyst (*n* = 86) and those with two cysts (*n* = 9). Of 104 DCs, 44 were central, 38 were lateral, and 22 were circumferential. DC types were not significantly affected by sex, age group, or anatomical location. Circumferential DCs often caused displacement of the mandibular canal inferiorly. While enucleation was preferred for the treatment of central DCs, circumferential DCs were treated with marsupialization.

**Conclusions:**

In this study, which is the first to evaluate the DC types on CBCT images, the central type was the most common. Circumferential DCs were mostly treated with marsupialization. CBCT imaging can assist in determining DC types, and may provide guidance for treatment planning.

## Background

A dentigerous cyst (DC) is a developmental cyst that originates from the dental follicle of an unerupted or impacted tooth, and encloses the tooth crown at the cementoenamel junction (CEJ) [1–4]. DCs occur in approximately 2.5–4% of patients with impacted teeth [3, 5]. They represent the second most common odontogenic cyst, accounting for 16–24% of all true cysts in the jaws [1–4].

While in most cases DCs involve permanent teeth, they may rarely be associated with unerupted primary teeth, supernumerary teeth, or odontomas [6].

Small lesions are generally asymptomatic and painless unless they become secondarily infected [5]. They may go unnoticed until they reach considerable size and cause pathological fractures [1, 2, 7]. They are often discovered incidentally during routine radiographic examination or when investigating the cause of a missing permanent tooth or delayed tooth eruption [2, 5, 7, 8].

Radiographically, a dentigerous cyst typically appears as a unilocular, round or oval-shaped radiolucent lesion attached to the CEJ, enclosing the crown of an unerupted tooth [2, 3, 5, 7, 8], with well-defined sclerotic margins [3, 9]. In infected cysts that are large enough to perforate the cortical bone, sclerotic margins may be lost, resulting in ill-defined borders [6]. While a normal follicular space is 2 to 3 mm, a DC can be suspected when the space exceeds 5 mm on 3D images [3].

Shear and Speight [2, 10] categorized DCs into three radiological types: central, lateral, and circumferential. In the central type, the crown of the tooth is symmetrically enveloped by the DC, while in the lateral type, the peri-coronal follicle expands only on one side of the crown. In the circumferential type, the entire tooth appears to be enclosed by the DC. It has been reported that DCs are predominantly of the central variety [11, 12].

To date, only one study has investigated the radiological types of DC in the maxilla and mandible using panoramic radiographs [13] based on Shear and Speight’s classification [2]. Three-dimensional (3D) imaging has been used to investigate the radiological types of DC only in the maxilla, and there is no comprehensive study in the literature that includes the DCs in the mandible.

To the best of our knowledge, this is the first study to examine the radiological types of DC using cone beam computed tomography (CBCT) according to the Shear and Speight’s classification in the international literature. The aim of this study was to investigate the radiological features and types of DC in the Turkish population, determine the prevalence and demographic characteristics of DCs, and the treatment methods used and review the relevant literature data.

## Methods

Approval for this study was obtained from the Institutional Review Board of Sivas Cumhuriyet University (No. 2023-11/33). The study was conducted in accordance to the principles laid out in the Declaration of Helsinki. Panoramic radiographs and CBCT images obtained from patients presenting to the Department of Oral and Maxillofacial Radiology at the Faculty of Dentistry, Sivas Cumhuriyet University between January 2020 and December 2023 who underwent biopsy and received treatment at the Department of Oral and Maxillofacial Surgery were included in this retrospective study.

The demographic data (age and sex), pathology reports, panoramic radiographs and CBCT images of patients diagnosed with DC were reviewed. The patients were divided into age groups of 0–9, 10–19, 20–29, 30–39, 40–49, 50–59, and 60–69 years.

Only patients with a histopathologically confirmed diagnosis of DC, complete clinical records, and high-quality images were included in the study.

Two patients with poor-quality CBCT images due to motion artifact, one patient with cleidocranial dysplasia having multiple impacted teeth and DCs, and six patients with only panoramic radiographs available and no CBCT images were excluded from the study. Ultimately, 95 patients diagnosed with DC, having both panoramic radiographs and CBCT images were included in the study.

With the significance criteria set at α = 0.05, β = 0.10, and 1-β = 0.90, the power of the test was estimated to be 0.9081 when 95 patients were included in the study [12].

### Display features

All panoramic and CBCT images were reviewed by a radiologist with 7 years of experience in maxillofacial radiology on an ASUS PB248Q Intel Core™ i7 monitor with a 32-bit LCD screen, 24.1-inch LED (light emitting diode) backlight, and a resolution of 1920 × 1200 pixels (ASUS, China) in a semi-lit room.

### Radiographic examination

Panoramic radiographs of all patients were obtained using a digital panoramic X-ray machine (Instrumentarium OP200 D, Instrumentarium Dental, Tuusula, Finland), and 3D images were acquired using a CBCT device (Planmeca ProMax 3D Mid, Planmeca Oy, Finland).

The number and location of the cysts identified in patients were examined. The locations of DCs were further divided into anterior, premolar, and molar regions in the maxilla and mandible. On panoramic radiographs, the region occupied by a large portion of the cystic lesion defined the lesion’s location.

**Fig. 1:**
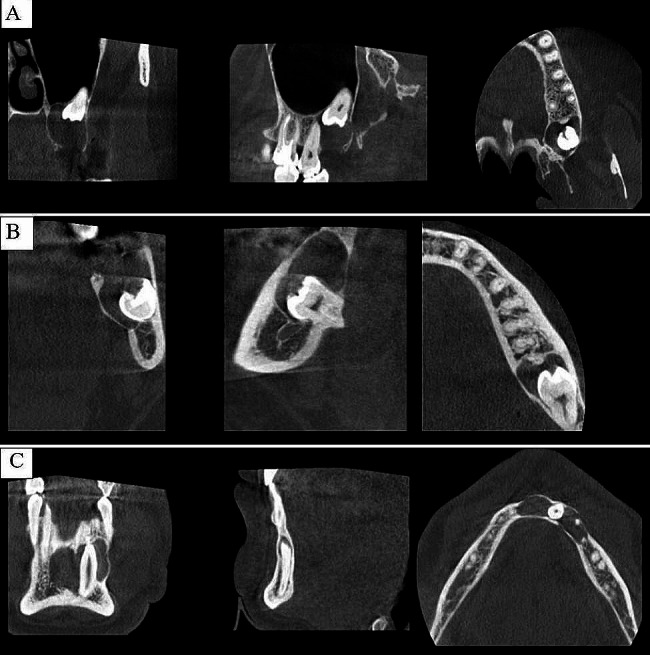
CBCT images of **(A)** Central, **(B)** Lateral, and **(C)** Circumferential Dentigerous Cysts in coronal, sagittal and axial planes

The radiological types of DC were evaluated according to Shear and Speight’s classification. DCs that appeared to symmetrically envelop the crowns of the associated teeth were classified as “central,” those expanding only on one side (mesial or distal) of the associated teeth were classified as “lateral,” and cysts that appeared to surround the entire associated tooth were classified as “circumferential” (Fig. 1).

DCs were classified into two categories based on their periphery: smooth and scalloped borders. Additionally, DCs were radiologically classified according to their shape as unilocular or multilocular.

The teeth associated with DC, changes in the affected/adjacent teeth (e.g., displacement, loss of lamina dura, or root resorption), and changes in the neighboring tissues (e.g., alterations in the mandibular canal, maxillary sinus, or inferior nasal border) were evaluated. Additionally, the presence of bone expansion (significant, minimal, or absent), opening of the cyst into the oral cavity due to cortical perforation, and the treatment method used were noted.

25% of the images were re-evaluated by the same researcher for intra-observer agreement two weeks later.

### Statistical analysis

The study data were analyzed using SPSS 23.0 (IBM Corp., Armonk, NY) software. Whether the data were normally distributed was checked using Shapiro-Wilk test. Mann-Whitney U test was used when comparing the measurements obtained from two groups. Chi-square and Fisher’s exact tests with contingency tables were used to compare categorical data. Kappa coefficients were used for intra-observer agreement, and the significance level was set at 0.05.

## Results

### Sex and age distribution

The mean age of the patients with DC (*n* = 95) was 33.97 ± 16.55 years (range, 8–64). 66 (69.5%) of the patients were male and 29 (30.5%) were female, with no significant difference between sexes in mean age (*P* = 0.108).

Among 95 patients, DCs were most commonly observed in the age group of 20–29 years (27.4%) and 40–49 years (18.9%) (*P* = 0.001).

When patients with single or two cysts were compared among age groups, both single and two cysts were more prevalent in the 20–29 and 40–49 age groups, but the difference was non-significant (*P* = 0.728) (Table 1).

**Table 1:** Sex and age distributions of dentigerous cysts

		Single DC (%)	Two DC (%)	Total (%)		
Sex	Male	60 (69.8%)	6 (66.7%)	66 (69.5%)	*P* = 0.558*	
	Female	26 (30.2%)	3 (33.3%)	29 (30.5%)		
	0 to 9 years	4 (4.7%)	0 (0.0%)	4 (4.2%)		
	10 to 19 years	14 (16.3%)	2 (22.2%)	16 (16.8%)		
	20 to 29 years	23 (26.7%)	3 (33.3%)	26 (27.4%)	X^2^ = 3.61	
Age Groups	30 to 39 years	9 (10.5%)	0 (0.0%)	9 (9.5%)	*P* = 0.728**	
	40 to 49 years	15 (17.4%)	3 (33.3%)	18 (18.9%)		
	50 to 59 years	14 (16.3%)	1 (11.1%)	15 (15.8%)		
	60 to 69 years	7 (8.1%)	0 (0.0%)	7 (7.4%)		
	Total	86(100.0%)	9 (100.0%)	95 (100.0%)		

*Fisher exact test (*P* < 0.05 denotes significance)

**Chisquare test, (*P* < 0.05 denotes significance)

In this study, a total of 104 DCs were found in 95 patients, including a single cyst in 86 (90.53%) and two cysts in 9 (9.47%) patients. Although DCs were more common in males than in females in patients with a single cyst or two cysts, no significant difference was found (*P* = 0.558) (Table 1).

### Localization of DCs

Of 104 DCs, the majority were observed in the mandibular molar teeth (57.7%), followed by the maxillary anterior teeth (17.30%), and the maxillary molar teeth (11.5%) (Table 2).

**Table 2:** Distribution of radiological types of dentigerous cysts by location

	Radiological type				
Location	Central	Lateral	Circumferential	Total	
Maxillary Anterior	10 (22.7%)	4 (10.5%)	4 (18.2%)	18 (17.3%)	X^2^ = 8.198 *P* = 0.610
Maxillary Premolar	1 (2.3%)	0 (0%)	0 (0.0%)	1 (1.0%)	
Maxillary Molar	6 (13.6%)	2 (5.3%)	4 (18.2%)	12 (11.5%)	
Mandibular Anterior	3 (6.8%)	4 (10.5%)	1 (4.5%)	8 (7.7%)	
Mandibular Premolar	1 (2.3%)	3 (7.9%)	1(4.5%)	5 (4.8%)	
Mandibular Molar	23 (52.3%)	25 (65.8%)	12 (54.5%)	60 (57.7%)	
Total	44 (100%)	38 (100%)	22 (100%)	104 (100%)	

*Chisquare test, (*P* < 0.05 denotes significance)

The most commonly involved teeth were mandibular third molars. Out of 73 DCs located in the mandible, 59 originated from third molars, 10 from canine teeth, 3 from second premolar teeth, and 1 from deciduous second molar teeth. Among the 31 DCs in the maxilla, 17 were associated with canine teeth, 11 with third molar teeth, and 1 each originated from second premolar tooth, first molar tooth or supernumerary incisor tooth.

### Radiological types and distribution of DCs

Among 104 DCs, 44 (42.31%) were of central type, 38 (36.54%) were of lateral type, and 22 (21.15%) were of circumferential type. No significant difference was found when DC types were compared between sexes and among age groups (*P* = 0.832 and *P* = 0.420, respectively). Despite the lack of significance among DC types in terms of location, a higher prevalence of all radiological types was observed in the mandibular molar region (*P* = 0.610) (Table 2).

Out of the 86 patients with a single DC, tooth-cyst relationship was central in 38, lateral in 31, and circumferential in 17. Among the 9 patients with two DCs each, 5 were central, 8 were lateral, and 5 were circumferential.

**Fig. 2:**
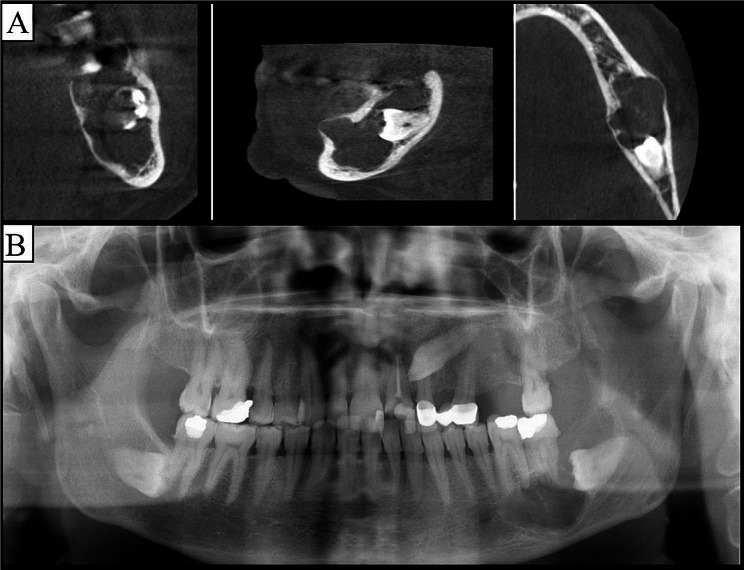
**(A)** CBCT image of a DC in the left mandibular region, with scalloped borders and partly well-defined margins in coronal, sagittal and axial planes, **(B)** Panoramic image of the same patient

All cysts were unilocular. The cyst margins were smooth in 94 (90.4%) DCs and scalloped in 10 (9.6%) DCs (Fig. 2). Smooth margin was significantly more common in all three DC types (*P* = 0.002).

Displacement of adjacent teeth was observed in 18 (17.3%) DCs, root resorption in 29 (27.88%) DCs, and loss of lamina dura in 55 (52.88%) DCs. Displacement was observed in 11(10.57%) of the impacted teeth.

In the mandible, it was found that circumferential DCs often caused displacement of the mandibular canal, while cysts of lateral and central types did not affect the canal (*P* = 0.01) (Table 4).

**Table 3:** Distribution of treatment methods by the type of dentigerous cyst and affected jaw

	Treatment method			
	Enucleation	Marsupialization	Total	
Central Lateral Circumferential	39 (50.0%) 30 (38.5%) 9 (11.5%)	5 (19.2%) 8 (30.8%) 13 (50.0%)	44 (42.3%)	X^2^ = 18.31 *P* = 0.001*
			38 (36.5%)	
			22 (21.2%)	
Mandibula Maxilla	50 (64.1%) 28 (35.9%)	23 (88.5%) 3 (11.5%)	73 (70.2%) 31 (29.8%)	X^2^ = 5.53 *P* = 0.019*
Total	78 (100%)	26 (100%)	104 (100%)	

*Chi-square test, (*P* < 0.05 denotes significance)

**Table 4:** Effect of mandibular dc types on mandibular canal

Effect on mandibular canal	Radiological type					
	Central	Lateral	Central		Total	
No change	18 (66.7%)	20 (62.5%)	3 (21.4%)	41 (56.2%)		X^2^ = 13.29
Inferior displacement	8 (29.6%)	7 (21.9%)	10 (71.4%)	25 (34.2%)		*P* = 0.010*
Perforation of canal	1 (3.7%)	5 (15.6%)	1 (7.1%)	7 (9.6%)		
Total	27(100.0%)	32(100.0%)	14(100.0%)	73(100.0%)		

*Chi-square test, (*P* < 0.05 denotes significance)

Out of the 31 maxillary DCs, 6 was associated with displacement of the maxillary sinus wall superiorly (4 central, 2 lateral), and 8 caused sinus perforation (4 central, 4 circumferential). The changes observed in the maxillary sinus wall were not significant for DC types (*P* = 0.207). A DC of lateral type caused displacement of the inferior nasal wall superiorly, while 4 cysts (3 circumferential and 1 central) perforated the inferior nasal border.

In 64 (61.53%) of the cysts, bone expansion was significant, while it was minimal in 28 (26.92%), and absent in 12 (11.53%). Although there was no significant association between radiological types and bone expansion, significant expansion was common in all types (*P* = 0.136).

Among 104 DCs, perforations of the buccal, lingual/palatal, alveolar crest, and inferior cortical bone (35 central, 34 lateral, 20 circumferential) were found in 89 (85.57%) cysts.

While 78 (75%) of the DCs were treated with enucleation, 26 (25%) were treated with marsupialization. In all enucleated cysts, the impacted teeth were removed. Enucleation was significantly more common in the cysts of central type, and marsupialization was more commonly preferred in circumferential DCs (*P* = 0.001). Both treatments were significantly more frequently used in the mandibular region (*P* = 0.019) (Table 3).

Kappa coefficients were classified as almost perfect (0.90-1), strong (0.80–0.90), moderate (0.60–0.79), weak (0.40–0.59), minimal (0.21–0.39), and no agreement (0.00-0.20) [14] In this study, intra-observer agreement ranged between nearly perfect and strong (*P* = 0.001) (Table 5).

**Table 5:** Results of statistical analysis for intra-observer agreement

	Intra- observer reliability (re-evaluating 25% of images) Kappa values	*P* value
Radiological type	0.940	0.001*
Radiological appearance	0.998	
Expansion	0.877	
Displacement of teeth	0.906	
Root resorption	0.866	
Loss of lamina dura	0.913	
Perforation	0.998	
Effect on mandibular canal	0.916	
Effect on maxillary sinus	0.873	
Effect on inferior nasal wall	0.998	

*Significant at *P* < 0.05

## Discussion

Using CBCT and panoramic images, this study reviewed the age and sex distribution of dentigerous cysts, their radiological features, types, locations, effects on anatomical structures, and treatment methods applied.

Across studies conducted on different populations, differences in the mean age have been observed; however, consistent with our findings, most studies have reported a higher frequency of DCs in males [2, 7, 13, 15, 16]. Although DCs were more commonly found in the 20–29 and 40–49 age groups in the current study, there was no significant age- or sex-related difference in the DC types (*P* = 0.832, *P* = 0.420, respectively). Noujeim et al. [13] reported a significant difference in the mean age of patients with lateral or central DCs (32.75 ± 2.3 years and 23.4 ± 1.6 years, respectively; *P* < 0.05), but in line with the current study’s findings, they did not find a relationship between DC types and sex. Differential results on the relationship between age and DC types across studies may be due to variations in the prevalence of cases that are often asymptomatic and therefore diagnosed late.

Patients with DCs rarely present with multiple cysts. Although multiple cysts were previously considered to be associated only with syndromes or systemic conditions such as mucopolysaccharidosis and cleidocranial dysplasia [17], cases not associated with a syndrome or systemic condition have also been identified [18, 19]. In the current study, 9.47% of the patients had two DCs each, and no association with any disease or syndrome was found. There was no significant difference between patients with single or two DCs with regard to sex or age groups (*P* = 0.558, *P* = 0.728). In a study on 109 Lebanese patients [13], multiple cysts were found in 22.9% of the patients (2 DCs in 22 patients and 3 DCs in 3 patients), and similar to the findings of the present study, no significant difference was observed between the two groups in terms of sex distribution and mean age. However, while multiple cysts were more common in males in the current study, they were more prevalent in females in that study [13]. This differential result may be attributed to differences in the characteristics of the populations studied. Further evaluation of the sex distribution of multiple cysts in a larger sample is warranted.

Regarding the location of DCs, it has been reported that DCs predominantly involve the mandible, with the mandibular third molars most commonly affected [13, 15, 16]. Other anatomical sites frequently involved include the maxillary canines, maxillary third molars, and mandibular second premolars [6, 20]. In line with the literature, no significant difference was found among the DC types in terms of location in the present study, and all three DC types were more commonly observed in the mandibular molar region (*P* = 0.633). In a published study, a significant association between DC types and jaw preference has been shown based on panoramic radiographs, with the lateral type found exclusively in the mandible and 89.7% of maxillary DCs being of the central type (*P* < 0.05) [13]. Another study evaluating only the maxilla using 3D imaging found that in 74 DCs involving a single tooth, the most common type was centripetal (47.3%), while the least common type was circumferential (12.16%) [21]. In that study, similar to the Shear and Speight’s classification, a DC was classified as the centripetal type if the cyst was located at the center of the tooth, as the eccentric type if the cyst was lateral to the long axis of the tooth or as the circumferential type if the cyst was partially attached to the apex of the root below the CEJ [21]. In the present study, where no significant difference was observed in DC types between the jaws (*P* = 0.059), a low prevalence of lateral DCs were observed in the maxilla. Minor variations observed in the reported rates of DC types across studies may be attributed to differences in imaging techniques, sample size or characteristics of the study population (e.g., race, ethnicity).

In contrast to studies classifying DCs into two types as central and lateral [22], Shear and Speight [2] reported a third variant known as the circumferential type. In the current study, based on CBCT examinations, the radiological types were central in 42.31%, lateral in 36.54%, and circumferential in 21.15% of 104 DCs according to Shear and Speight’s classification. Using the same classification, Noujeim et al. [13] reported that among 137 DCs, the central type was the most common (60.6%), followed by the lateral type (29.2%), and the circumferential type (10.2%). The higher prevalence of the central type found in that study [13] may have resulted from evaluation of the DCs on two-dimensional (2D) images in the aforementioned study. Bucco-lingual expansion that cannot be visualized on 2D panoramic radiographs may cause inaccurate differentiation among the radiological DC types. Furthermore, in a study using CBCT images, which classified 18 DCs into two types (central and lateral), 56% of the cysts were of the central type and 44% were of the lateral type [22], with a similarly high prevalence of the lateral type as observed in the present study. However, the circumferential type was not evaluated in that study.

In parallel with our findings, previous studies [11, 21–23] have reported that the majority of DCs appear as unilocular, expansive lesions with smooth margins on radiographs. DCs that reach a considerable size may exhibit a scalloped appearance [23, 24]. Although all of the cysts evaluated in the present study were unilocular and often expansive, DCs with a scalloped appearance were also observed. Former studies have reported DCs with a multilocular appearance on 2D images [23]. In those studies, some cysts with a scalloped appearance may have been misinterpreted as being multilocular. Therefore, the use of CBCT images would be more reliable. CBCT provides important information for the differential diagnosis of DCs, such as changes in affected teeth such as resorption, and the extent of cortical expansion [22]. Three-dimensional images can provide precise information about the size, location, content, and relationships of the lesions, and therefore, they are more relevant and valuable for planning appropriate treatment than plain radiographs [25]. CBCT is also useful in the evaluation of a cystic lesion expanding in the bucco-lingual direction or to determine the relationship of the cyst to the complex anatomy of the maxilla [10]. Thorough assessment of large DCs with 3D imaging, giving particular attention to the CEJ, can provide valuable information for differential diagnosis. DCs may be considered in the preliminary diagnosis of lesions, especially those originating at the CEJ level. Other important lesions that should be considered in the differential diagnosis include odontogenic keratocyst, orthokeratinized odontogenic cyst and ameloblastoma. These arise from the epithelial remnants of the dental lamina derived from connective tissue lining the dental follicle, and may embrace the tooth as the cyst or tumor enlarges [10]. In addition, adenomatoid odontogenic tumor and calcified odontogenic cysts, which are often associated with an impacted tooth (usually an upper canine) should be considered in the differential diagnosis. Both of these lesions may enclose the tooth crown and root. Compared to DCs, odontogenic keratocysts show less expansion to the bone and are less likely to cause root resorption [3, 10]. In the current study, the frequency of root resorption in adjacent teeth was 29 (27.88%). It is challenging for clinicians to distinguish the circumferential type DCs that surround the tooth from other lesions, and DC should be borne in mind in the differential diagnosis even if the lesion extends beyond the CEJ. In such cases, histopathological examination is crucial [10].

Dentigerous cysts may cause changes in the mandibular canal, maxillary sinus, and the base of the nasal fossa [3]. In the present study, it was shown that 24.5% of the 73 mandibular cysts caused displacement of the mandibular canal inferiorly, while 6.86% perforated the canal. When the effect of the DC types on the mandibular canal was investigated, it was observed that circumferential type DCs displaced the mandibular canal inferiorly, while lateral and central cysts did not have an effect on the canal (*P* = 0.010). In a study conducted on a small sample [22], displacement of the mandibular canal was reported in 5 out of 12 (42%) mandibular DC cases [22]. The significant effect of the circumferential cysts on the mandibular canal as observed in the current study may be explained by their particular tendency to grow towards the apex of the tooth and more often reaching larger dimensions compared to other types.

In the present study, when the relationship between the lesions and the maxillary sinus wall was evaluated, displacement of the maxillary sinus wall was observed in 19.35%, while perforation of the maxillary sinus wall was found in 25.8% of 31 cysts. There was no significant relationship between DC types and their effect on the maxillary sinus (*P* = 0.207). The nasal floor was affected in 5 cases (16.1%). Although there was a greater number of cysts in the mandible, the anatomical structures of the maxilla were more likely to be affected by the cysts. This can be explained by the fact that bone density is lower in the maxilla, allowing the cysts to expand more readily. In a study, it was reported that out of 79 maxillary DCs, 34 (43%) affected the maxillary sinus, and 45 (57%) affected the boundary of the nasal fossa [21]. In the same study, 26 of the maxillary cysts were partially and 8 were entirely located in the maxillary sinus, but maxillary sinus displacement was not evaluated. Regarding the nasal floor, it was reported that 5 cysts involved the nasal floor without impairing its integrity, while 40 cysts caused elevation or discontinuation of the nasal floor. The higher prevalence rates reported in previous studies compared to the present study may be due to differences in the number of DCs involving the anterior maxillary region.

In the treatment of DCs, orthodontic intervention can be performed on affected teeth, cysts can be enucleated with or without removal of the tooth involved, or marsupialization can be applied to large cysts [6, 26]. However, DCs are usually treated with enucleation [3]. In the present study, most cysts were enucleated, with enucleation being more frequently used for central DCs and marsupialization preferred for circumferential DCs (*P* = 0.001). In particular, circumferential DCs were often marsupialized to preserve various anatomical structures and prevent paresthesia due to their tendency to reach a larger size.

A limitation of this study was the inability to investigate a number of clinical parameters such as pus discharge from the lesion, paresthesia, and pain in the affected region due to incomplete documentation.

## Conclusion

This study was the first to evaluate the types of dentigerous cysts using CBCT images in a Turkish sample, and discussed the potential benefits of 3D imaging, highlighting the characteristics of DC types and their impact on surrounding tissues. The central type was the most common variant, followed by lateral, and circumferential types. The numbers and types of DC were not affected by age, sex, or anatomical site. Circumferential DCs may cause displacement of the mandibular canal, and they are generally treated with marsupialization. Central DCs, on the other hand, are usually enucleated. The radiological types of DC as determined by Shear and Speight’s classification on CBCT images can provide guidance for the treatment approach to be used, thereby facilitating the clinical decision-making process.

## Data Availability

The datasets created and/or analyzed during the current study are not publicly available due to [Ethics committee decision], but are available from the corresponding author upon reasonable request.
